# Curcumin Attenuation of Wear Particle-Induced Osteolysis via RANKL Signaling Pathway Suppression in Mouse Calvarial Model

**DOI:** 10.1155/2017/5784374

**Published:** 2017-09-20

**Authors:** Tao Cheng, Yaochao Zhao, Bin Li, Mengqi Cheng, Jiaxing Wang, Xianlong Zhang

**Affiliations:** ^1^Department of Orthopaedic Surgery, Shanghai Jiao Tong University Affiliated Sixth People's Hospital, Shanghai, China; ^2^Department of Orthopaedic Surgery, Renji Hospital, Shanghai Jiao Tong University School of Medicine, Shanghai, China; ^3^Department of Orthopaedic Surgery, The First Affiliated Hospital of Zhengzhou University, Zhengzhou, Henan, China

## Abstract

Wear particle-induced chronic inflammation and osteoclastogenesis are two critical factors in the osteolytic process. Curcumin (CUR) is an active compound of the medicinal herb *Curcuma longa* and has anti-inflammatory and antiosteoclastogenic properties. Our study tested the hypothesis that CUR might attenuate polymethylmethacrylate- (PMMA-) induced inflammatory osteolysis using mouse calvaria osteolysis model in vivo and in vitro. The mice were divided into four groups: phosphate-buffered saline group, CUR, PMMA, and PMMA + CUR groups. Three days before PMMA particle implantation, the mice were intraperitoneally injected with CUR (25 mg/kg/day). Ten days after the operation, the mouse calvaria was harvested for microcomputed tomography, histomorphometry, and molecular biology analysis. As expected, CUR markedly reduced the secretion of tumor necrosis factor-*α*, interleukin- (IL-) 1*β*, and IL-6 in the calvarial organ culture. Moreover, CUR suppressed osteoclastogenesis and decreased bone resorption in vivo compared with PMMA-stimulated calvaria. Furthermore, CUR downregulated the osteoclast-specific gene expression and reversed the receptor activator of nuclear factor kappa-B ligand (RANKL)/osteoprotegerin messenger RNA and protein ratio in PMMA particle-stimulated mice. These results suggest that CUR attenuated PMMA particle-induced inflammatory osteolysis by suppressing the RANKL signaling pathway in the murine calvarium, which could be a candidate compound to prevent and treat AL.

## 1. Introduction

Hip arthroplasties are among the most successful procedures for end-stage joint diseases. However, periprosthetic osteolysis and subsequent aseptic loosening (AL) are the main causes of implant failure and affect approximately one-third of patients 10–20 years postoperatively [1–4]. Wear particles generated at joint articulations and nonarticulating interface play critical roles in periprosthetic osteolysis pathogenesis [2, 5, 6]. Currently, revision surgery for wear particle-induced osteolysis is the only effective treatment for AL. Unfortunately, it is associated with high morbidity, mortality, complication rates, and poor functional outcomes [1, 5]. Recently, the majority of research in relation to AL has focused on the mechanisms involved in the modulation of inflammation and differentiation of osteoclast precursors into mature osteoclasts at the molecular level through pharmacological intervention [6–10].

The pathogenesis of periprosthetic osteolysis involves the production of proinflammatory cytokines, osteoclast activation, and bone loss after wear particle stimulation [1, 6]. The activation of the receptor activator of nuclear factor kappa-B (RANK)/RANK ligand (RANKL) axis is associated with osteolysis adjacent to bone-implant interfaces [5, 11]. The modification of nuclear factor-kappa B (NF-*κ*B) signaling pathway is a potential therapeutic target for wear particle-induced osteolysis [9, 10, 12, 13].

Curcumin (CUR), which is isolated from the root of the medicinal plant *Curcuma longa*, is a biocompatible and biodegradable polyphenolic compound [14–17]. It may protect against ovariectomy-induced bone loss and strengthen the bones [15, 16]. The inhibitory effect of CUR on osteoclastogenesis occurs through RANK/RANKL signaling impairment. Moreover, CUR may attenuate inflammatory reactions by inhibiting proinflammatory mediators (tumor necrosis factor- (TNF-) *α* and interleukin- (IL-) 1*β*) and modulate cellular immune responses by suppressing NF-*κ*B activation [14, 17]. These factors contribute to osteoclast activation and final bone resorption. Our study suggested that CUR inhibits titanium particle-induced inflammatory responses in a mouse air-pouch model [18]. Based on previous studies [17, 18], we proposed that CUR is a potential candidate for prophylactic treatment against periprosthetic osteolysis and AL. However, whether the antiresorptive capabilities of CUR are associated with the RANKL/osteoprotegerin (OPG) signaling system during particle-induced osteolysis remains unclear.

The present study aimed to investigate the effects of CUR on polymethylmethacrylate (PMMA) particle-induced osteolysis, as well as osteoclastogenic signaling pathways involved, using the murine calvarial model in vivo and in vitro.

## 2. Materials and Methods

### 2.1. Preparation of PMMA Particles and CUR

Commercially available PMMA particles (Polysciences) with a mean diameter of 4.8 *μ*m (0.1–16) were used in this study as described previously [19]. Ninety percent of the particles were <10 *μ*m in diameter. Adherent endotoxin in particles was removed by sterilization in 70% ethanol for 48 h and washing with sterile phosphate-buffered saline (PBS) thrice. The absence of endotoxin in PMMA particles was confirmed using a Limulus Amebocyte Lysate Kit (BioWhittaker, Walkersville, MD) at a detection level of <0.05 EU/ml. The PMMA particles were then suspended in sterile PBS at a concentration of 1 mg/ml. CUR with ≥98% purity was obtained from Aladdin Industrial Incorporation (Ontario, CA, USA). A total of 1 mg CUR was dissolved in 0.5 ml dimethyl sulfoxide (DMSO) at a concentration of 2 mg/ml and stored at −20°C until needed.

### 2.2. Animal Model and Study Design

The experimental design was approved by the Laboratory Animal Care and Use Committee of Shanghai Jiao Tong University. Female BALB/c mice, aged 9-10 weeks, were purchased from the Shanghai Laboratory Animal Center (Chinese Academy of Sciences). As previously described [10, 13, 20], a murine calvarial model of PMMA particle-induced osteolysis was used in this study. Briefly, 50 *μ*l of PMMA particle suspensions were injected under the periosteum around the sagittal middle suture of the mice to induce osteolysis after the mice were anesthetized using 0.25 ml 4% chloral hydrate via intraperitoneal injection. A total of 72 healthy mice were randomly divided into four groups: (1) PBS (phosphate-buffered saline) group (negative control group), where the mice underwent sham operation and received PBS treatment; (2) CUR group, where the mice underwent sham operation and received CUR treatment; (3) PMMA group (positive control group), where the mice received PMMA particles; and (4) PMMA + CUR group (therapeutic intervention group), where the mice received PMMA particles plus CUR. Mice were injected intraperitoneally with CUR (25 mg/kg) daily, beginning three days prior to PMMA particle implantation. Positive control mice were injected with same volumes of DMSO in PBS in the same schedule. Ten days after the procedure, the mice were euthanized, and calvarias were dissected and harvested for further molecular and histological analyses. The mice had free access to food and water. No adverse effects or fatalities were noted during the experimental period.

### 2.3. Microcomputed Tomography (*μ*CT) Imaging and Volumetric Osteolysis Analysis

After sacrificing six mice per group 10 days after wear particle stimulation, their calvarial bones were harvested and fixed in 10% formaldehyde for three days. Then, the entire calvarias were scanned and analyzed using three-dimensional (3D) *μ*CT system (Skyscan 1072, Skyscan, Aartselaar, Belgium) at a resolution of 10 *μ*m. After 3D image reconstruction, a cylinder region of interest (3 mm in diameter and 1 mm thick) around the sagittal midline suture was selected for quantitative analyses, including bone mineral density (BMD, mg/cc), bone volume/tissue volume (BV/TV), number of porosities, and percentage of total porosity measured, according to previous literature [10, 13].

### 2.4. Histological Analysis and Tartrate-Resistant Acid Phosphatase (TRAP) Staining

After *μ*CT scanning, the harvested calvarias were decalcified in 10% EDTA (pH 7.4) for three weeks at 4°C and were processed for paraffin embedding using standard method. The tissue sections (4 *μ*m) cut in the coronal plane using a microtome were prepared for hematoxylin and eosin (H&E) and Masson's trichrome staining. Histomorphometric analysis was performed using Image-Pro image analysis software package (Media Cybernetics, MD, USA) as described previously [13, 20]. Osteoclast formation was determined by TRAP staining using a histochemical kit (Sigma, St. Louis, MO). The dark purple-staining granules in the cytoplasm were considered a specific criterion for TRAP-positive (TRAP+) cells. To evaluate bone resorption, the ratio of the remaining area of the bone (RRAB, %), eroded surface area (ESA, mm^2^), number of TRAP+ cells, and percentage osteoclast surface per bone surface (OcS/BS, %) were determined in the round region of interest (3 mm in diameter) of the five consecutive sections, as described previously [13, 21].

### 2.5. Gene Expression Analysis

The total RNA of tissue samples was extracted using Trizol reagent (Invitrogen, Carlsbad, CA) according to the manufacturer's instructions. Real-time reverse transcription polymerase chain reaction (RT-PCR) was used to quantify messenger RNAs (mRNAs). First-strand complementary DNA (cDNA) was synthesized using M-MLV reverse transcriptase and cDNA synthesis kit (Takara, Tokyo, Japan). Subsequently, 1.0 *μ*l cDNA from each sample was used for PCR reactions using a SYBR green kit (Takara, Tokyo, Japan) and a Thermal Cycler Dice TP800 (Takara Bio, Kyoto, Japan). The forward and reverse primer sequences for TRAP, calcitonin receptor (CR), cathepsin K (CK), nuclear factor of activated T cells c1 (NFATc1), OPG, RANKL, and the housekeeping gene GAPDH were used as described in our previous studies [9, 13, 22].

### 2.6. ELISA Analysis of Cultured Calvaria

The entire murine calvaria (6 per group) were dissected under sterile conditions and cultured with Dulbecco's modified Eagle's medium (DMEM) with 1% penicillin and streptomycin for 24 h at 37°C with 5% CO_2_. The culture medium was harvested and stored at −80°C to check for TNF-*α*, IL-1*β*, IL-6, OPG, and RANKL secretion using ELISA kits (R&D, Minneapolis, MN).

### 2.7. Liver and Kidney Function Tests

The blood samples were collected at the end of the experiment and centrifuged (1500g) at 4°C for 15 min. To monitor for possible renal toxicity or hepatotoxicity of CUR in animals, the serum levels of alanine transaminase (ALT), aspartate transaminase (AST), creatinine (Cre), and blood urea nitrogen (BUN) were measured using diagnostic kits (Nanjing Jiancheng Biotech, Nanjing, China) according to the manufacturer's instruction.

### 2.8. Statistical Analysis

Data were presented as mean ± SD. Student's *t*-test was used to determine the statistical significance between groups. A *p* value of <0.05 was considered statistically significant.

## 3. Results

### 3.1. Effect of CUR on PMMA Particle-Induced Osteolysis

**Figure 1:**
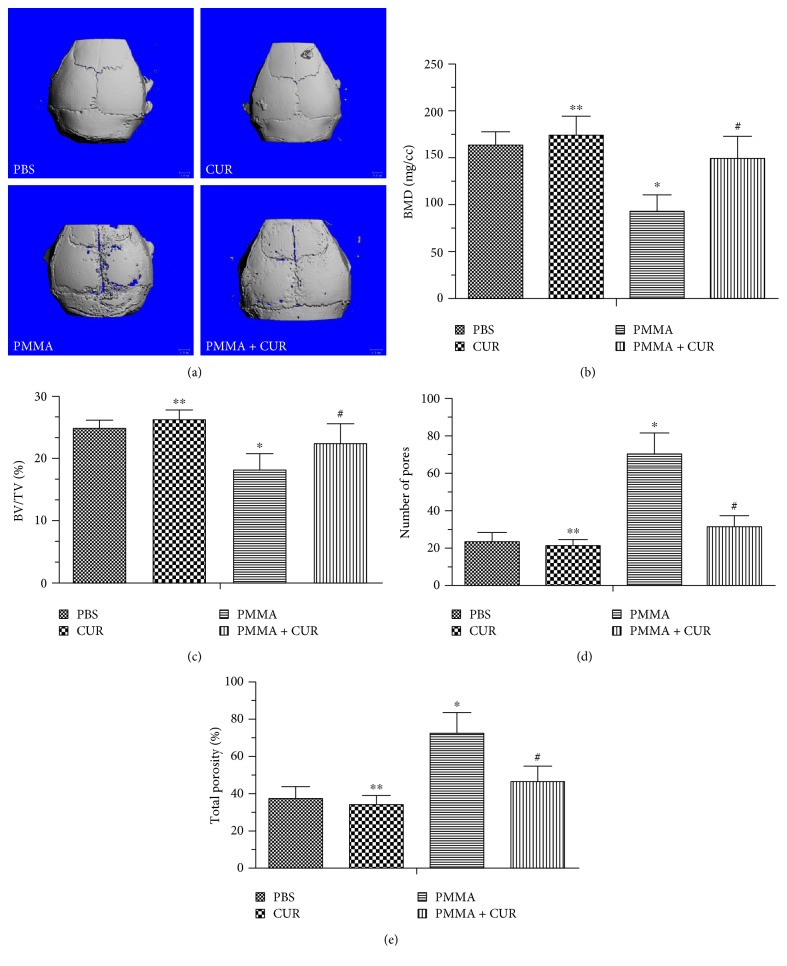
Curcumin prevented PMMA particle-induced mouse calvarial osteolysis. (a) Representative 3D *μ*CT reconstructed images of the calvaria in each group (*N* = 6/group). Measured (b) BMD, (c) trabecular BV/TV ratio, (d) number of pores, and (e) the percentage of total porosity within the ROI from each sample (*N* = 6/group; ^∗∗^*p* > 0.05 versus the PBS group; ^∗^*p* < 0.001 versus the PBS group; ^#^*p* < 0.01 versus the PMMA group).

The *μ*CT image revealed that particle implantation elicits localized inflammatory and osteolytic responses that significantly widened the cranial suture width and bone porosity in the untreated mice (PMMA group) compared with the PBS group. Conversely, sham operation groups without particle stimulation showed no pronounced osteolysis (Figure 1(a)). As expected, a marked increase in bone volume and pronounced reduction of cranial suture width were observed in the CUR group compared with that of the untreated group. According to the 3D image, the PMMA group revealed a significant decrease in all four parameters (BMD, BV/TV, number of porosities, and percentage of total porosity) compared with the PBS group (*p* < 0.001; Figures 1(b), 1(c), 1(d), and 1(e)). By comparison, CUR treatment attenuated the osteolytic bone destruction and reversed the reduced bone volume and increased bone porosity (*p* < 0.01; Figures 1(b), 1(c), 1(d), and 1(e)). The bone structure parameters all trended toward improved structure in the CUR-treated mice in the sham group than that of the negative control group. However, these differences were not statistically significant (*p* > 0.05; Figures 1(b), 1(c), 1(d), and 1(e)).

**Figure 2:**
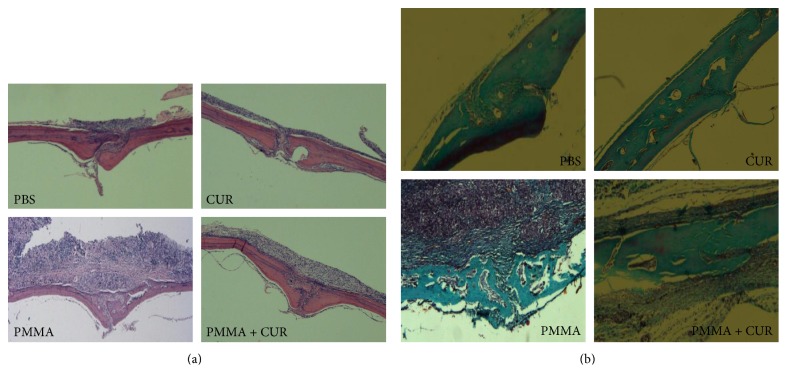
Curcumin prevented PMMA particle-induced mouse calvarial osteolysis. (a) Representative hematoxylin and eosin (H&E) staining of calvarial sections in each group (*N* = 6/group). (b) Representative Masson trichrome staining of calvarial sections in each group (*N* = 6/group).

**Figure 3:**
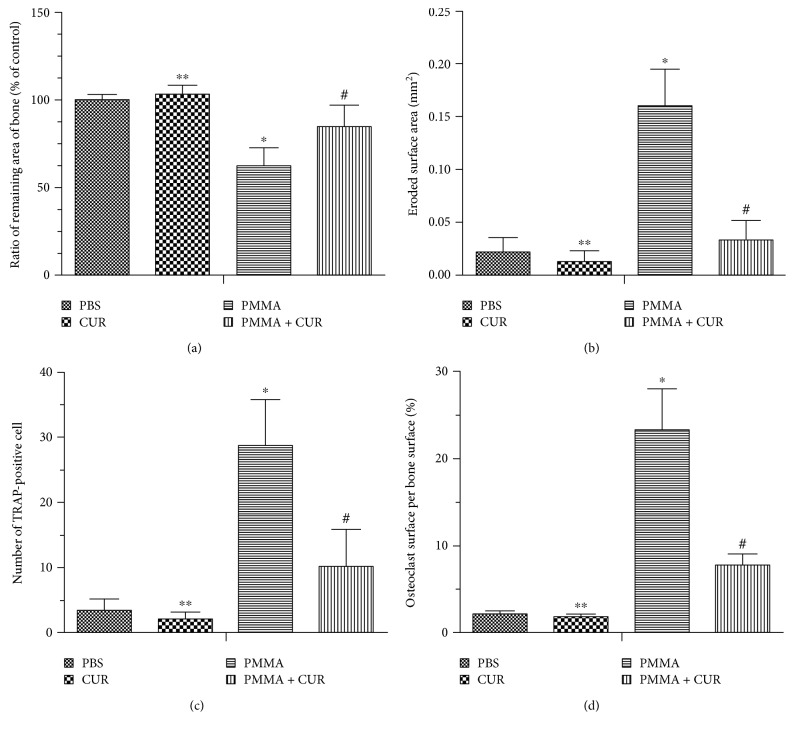
Curcumin prevented PMMA particle-induced osteoclastogenesis. Histomorphometric analysis of (a) the ratio of remaining area of bone (%), (b) the eroded surface area (mm^2^), (c) the number of TRAP-positive multinucleated osteoclasts, and (d) the percentage of osteoclast surface per bone surface (OcS/BS, %) within the ROI in each group (*N* = 6/group; ^∗∗^*p* > 0.05 versus the PBS group; ^∗^*p* < 0.001 versus the PBS group; ^#^*p* < 0.01 versus the PMMA group).

As shown in Figure 2, H&E and trichrome staining demonstrated that trabecular bone was well conserved with a faint inflammatory reaction in the sham-surgery groups. The tissues exposed to PMMA particles for 10 days showed a marked inflammatory reaction and extensive bone erosion with numerous macrophages and multinucleated giant cells. The bone resorption of the mice receiving CUR treatment (PMMA + CUR group) was significantly reduced compared with that of the mice in the positive control group (PMMA group) on the RRAB and ESA parameters (*p* < 0.01; Figures 3(a) and 3(b)).

### 3.2. Effect of CUR on PMMA Particle-Induced Osteoclastogenesis

TRAP staining was applied in assessing the osteoclast amount to determine whether CUR attenuated PMMA particle-induced osteoclastogenesis in vivo. The results revealed that TRAP+ cells were present along the eroded bone surface in the PMMA group. CUR treatment significantly reduced the number of TRAP+ cells and OcS/BS in the particle-implanted mice compared with the PMMA group (*p* < 0.01, Figures 3(c) and 3(d)). No difference was detected in TRAP staining between the PBS only group and CUR group (*p* > 0.05, Figure 3(c)).

### 3.3. Effects of CUR on the Expression Levels of Osteoclast-Specific Gene Markers and RANK Ligand

**Figure 4:**
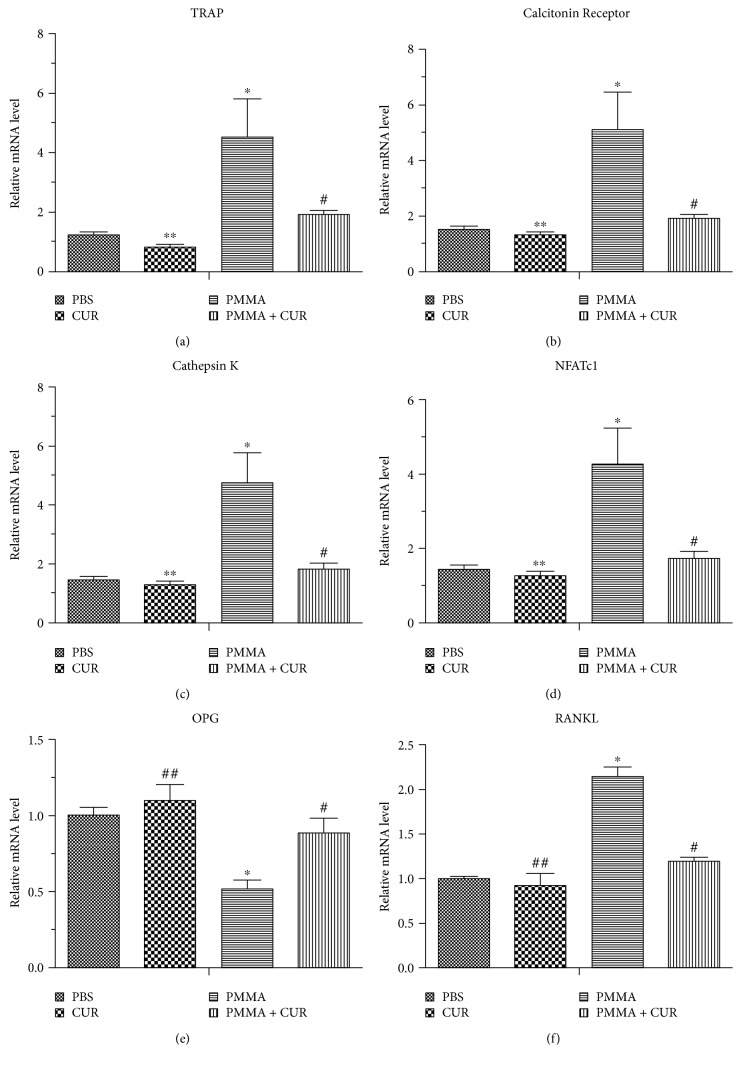
The mRNA levels of TRAP, CTR, CK, NFATc1, OPG, and RANKL were analyzed by RT-PCR, and the results were normalized to the expression in the PBS groups (*N* = 6/group; ^∗∗^*p* > 0.05 versus the PBS group; ^##^*p* < 0.05 versus the PBS group; ^∗^*p* < 0.001 versus the PBS group; ^#^*p* < 0.01 versus the PMMA group).

RT-PCR was used to assess the mRNA expression levels of TRAP, CR, CK, NFATc1, OPG, and RANKL in the calvaria tissue. As depicted in Figure 4, mice challenged with PMMA particles showed increased levels of gene expression for osteoclast-specific marker and RANKL, whereas OPG mRNA expression levels were markedly decreased compared with that of the negative control group (*p* < 0.001). As expected, the numbers of the gene copy of osteoclast-specific marker and RANKL in mice implanted with PMMA particles were significantly reduced by CUR treatment (*p* < 0.01). Moreover, the mRNA expression of OPG was significantly upregulated in the therapeutic group (CUR group) compared with that of the PMMA group (*p* < 0.01).

### 3.4. Effects of CUR on the Secretion Levels of Cytokines and RANK Ligand

**Figure 5:**
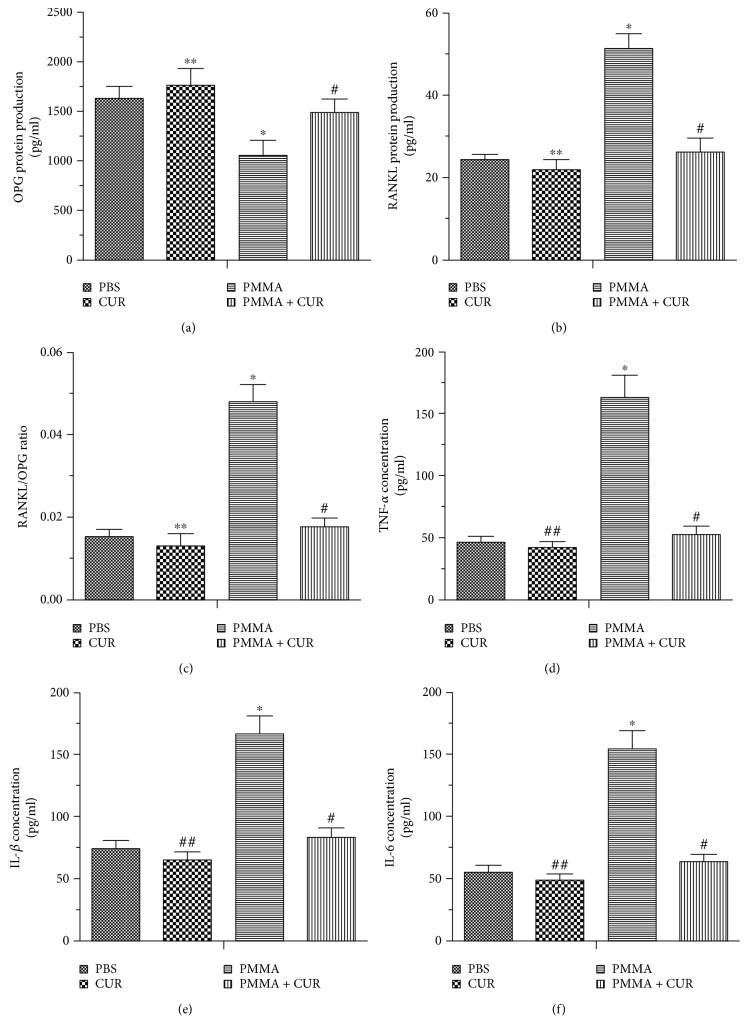
ELISA results for TNF-*α*, IL-1*β*, IL-6, OPG, RANKL protein levels, and the RANKL/OPG ratio in the supernatants of cultured calvaria (*N* = 6/group; ^∗∗^*p* > 0.05 versus the PBS group; ^##^*p* < 0.05 versus the PBS group; ^∗^*p* < 0.001 versus the PBS group; ^#^*p* < 0.01 versus the PMMA group).

We developed an organ culture system in vitro and evaluated the protein levels of cytokines and RANK ligand in the DMEM of cultured calvaria using ELISA. PMMA particles significantly inhibited OPG release, but markedly increased the release of TNF-*α*, IL-1*β*, IL-6, and RANKL in the medium compared with the PBS group (*p* < 0.001; Figures 5(a), 5(b), 5(d), 5(e), and 5(f)). The OPG secretion in the PMMA + CUR group was higher than that of the PMMA group, whereas the protein levels of TNF-*α*, IL-1*β*, IL-6, and RANKL were suppressed markedly by CUR treatment (*p* < 0.01; Figures 5(a),5(b), 5(d), 5(e), and 5(f)). Moreover, PMMA particles significantly increased the relative ratio of RANKL/OPG compared with the PBS group (*p* < 0.001; Figure 5(c)). However, CUR administration reversed the imbalance in the RANKL/OPG ratio after particle stimulation (*p* < 0.01; Figure 5(c)).

### 3.5. Determination of Liver and Kidney Function

Liver and renal function tests were performed to assess CUR-induced liver and kidney damage. As shown in Table 1, the serum levels of ALT, AST, Cre, and BUN were not statistically different between the CUR and PBS groups (*p* > 0.05).

## 4. Discussion

Despite numerous studies about AL, effective prevention and treatments remain limited [1, 6]. CUR is a major active component derived from the spice turmeric and purportedly has diverse biological effects that are anti-inflammatory, antioxidant, antiviral, and anti-infectious. In the current study, we confirmed the effects of CUR on the expression levels of proinflammatory cytokines and osteoclastogenesis in mouse calvaria tissues and explored the mechanisms of CUR in reducing inflammatory osteolysis after PMMA particle implantation. AL is initiated by local inflammatory response to implant-derived wear particles that activate and recruit macrophages and osteoclasts, thereby leading to fibrous tissue interface and periprosthetic bone resorption. Consistent with the 3D *μ*CT scanning, histomorphometric and histological assessments demonstrated that osteolytic suppression was accompanied by decreased gene expression levels of osteoclast-specific markers and TRAP+ osteoclasts in the CUR treatment group after PMMA particle stimulation. Our previous study demonstrated that CUR suppresses titanium particle-induced inflammation by regulating macrophage polarization [18]. Therefore, these above results support the present hypothesis that CUR might be a natural compound that prevents and treats periprosthetic osteolysis and AL.

Active bone resorbing osteoclasts are critical for the development of bone resorption. Therefore, investigating CUR to mitigate wear particle-induced osteoclastogenesis effectively is crucial for treating osteolysis. We found that PMMA particles caused marked bone loss and that a large number of osteoclasts are present in osseous tissue in murine calvaria using *μ*CT and TRAP staining. Moreover, CUR treatment effectively increased bone volume and decreased TRAP+ osteoclasts induced by PMMA particles. These findings indicate that CUR plays a positive role in preventing particle-induced osteolysis related to osteoclasts. Several recent studies have demonstrated that CUR abrogates osteoclast differentiation and function and provides a useful bone-protecting drug in treating postmenopausal osteoporosis [15, 23, 24].

RANKL, a member of the TNF family, supports osteoclast activation, differentiation, and maturation [4, 25]. Moreover, OPG is a naturally occurring decoy receptor for RANKL and downregulates osteoclast-specific gene expression and osteoclastogenesis by binding with RANKL, thereby preventing interaction with RANK [4, 25]. Previous studies have indicated that the ratio of RANKL and OPG expressions in the interface membrane or synovial fluid of patients is critical to the occurrence of periprosthetic osteolysis [4, 25]. A recent study using RANK knockout mice has proven that RANK/RANKL signaling pathway is essential for wear particle-induced bone resorption [26]. Exogenous OPG gene modification effectively suppresses particle-induced osteolysis and regains implant stability using a murine model [27]. Recent studies have found that CUR decreases osteoclastogenesis by suppressing the RANK/RANKL system [23, 24]. We consistently found increased amount of protein and mRNA expression levels of OPG after CUR administration. Moreover, RANKL expression in the CUR treatment group was reduced compared with that of the positive control group in the presence of PMMA particles. The mechanisms of the inhibitory effects of CUR were mediated via the suppression of the RANKL/RANK signaling pathway, which contributed to the inhibition of osteoclast formation and treatment of particle-induced osteolysis.

Our study revealed that CUR slightly promoted bone formation in calvaria in terms of bone mass parameters, although the differences were not statistically significant between the mice in the sham groups without PMMA particle implantation. Chen et al. [28] found that CUR could ameliorate glucocorticoid-induced osteoporosis by protecting osteoblasts from apoptosis. Hussan et al. [29] reported that high dosage of CUR could protect against ovariectomy-induced bone loss by increasing the number of osteoblast in rat models. Our results are consistent with previous observations regarding the bone sparing effects of CUR. However, CUR treatment was given intragastrically for two months in Hussan's study [29], whereas CUR was administered intraperitoneally for only two weeks in our study. Thus, further research is needed to explore the dose, timing, and route of drug administration.

Phagocytosis of wear particles elicits inflammatory reactions and release of proinflammatory cytokines that contribute to osteoclast activation and subsequent bone resorption. Moreover, CUR exhibits anti-inflammatory activities on collagen-induced arthritis [17]. To test the hypothesis that CUR can effectively suppress particle-induced inflammatory response by inhibiting proinflammatory mediators. Our data confirm that CUR significantly inhibits the production of TNF-*α*, IL-1*β*, and IL-6, which are the main cytokines in the interface membrane. Therefore, we propose that the inhibitory effects of CUR on proinflammatory cytokines can potentially reduce chronic inflammation by wear particles.

To the best of our knowledge, this is the first experiment that confirms that CUR can inhibit PMMA particle-induced osteolysis by regulating the RANKL/OPG signaling system. However, this present study has several limitations that should be considered. First, commercially available PMMA particles were used in a murine calvarial model rather than other types of wear particles, such as ultrahigh molecular weight polyethylene particles, alumina ceramic particles, and cobalt chromium particles, which are seen in periprosthetic tissues. The osteolytic process can be influenced by the type, size, shape, and concentration of wear particles [2, 3, 30, 31]. Therefore, further investigation is encouraged to determine particle bioactivity. Second, the CUR pharmacokinetic evidence is not provided in the present study. Thus, the clinical relevance of our observations concerning CUR in AL remains unknown. Previous clinical trials have shown that oral administration of CUR relieved pain and improved function in osteoarthritis and rheumatoid arthritis patients [32–34]. Issues remain over the relatively poor bioavailability of oral administration [35]. To overcome this problem, we tried dissolving CUR in DMSO that is widely available in the clinical and experimental applications as a solvent [36–38]. The liver and kidney injuries were measured with ALT, AST, Cre, and BUN. No signs of toxicity and major side effects in animals receiving CUR treatment were observed in the present study. Therefore, CUR was injected intraperitoneally in mice without major side effects. Third, the small-animal model (murine calvarial osteolysis model) was not completely equal to the chronic osteolytic process of AL due to the lack of implant, mechanical load, and continuous particle infusion. To identify the long-term effectiveness and safety of CUR treatments, we need to develop large-animal models with prosthesis implantations. In the past decades, murine calvarial model has been among the most common animal models for investigating complex cellular and tissue mechanisms involved in the process of wear debris-associated bone resorption [13, 20, 39]. Despite these limitations, the results of our study using the murine model can provide valuable data and facilitate preclinical testing of CUR treatment.

## 5. Conclusions

**Figure 6:**
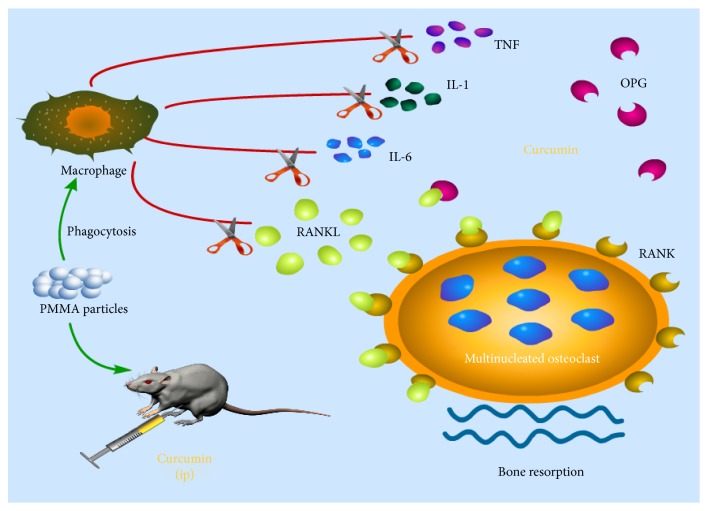
Schematic representation of the molecular mechanisms of curcumin action in particle-induced osteolysis. CUR markedly reduced the secretion of inflammatory cytokines. Furthermore, CUR downregulated the osteoclast-specific gene expression and reversed the receptor activator of nuclear factor kappa-B ligand (RANKL)/osteoprotegerin ratio in PMMA particle-stimulated mice.

In conclusion, the present study shows that prophylactic treatment with CUR attenuates PMMA particle-induced inflammatory osteolysis in murine calvaria in vitro and in vivo. The inhibitory effects of CUR on osteoclast-specific genes and osteoclastogenesis occur through the enhanced OPG and deletion of RANKL/RANK signaling pathways. Furthermore, we observed that proinflammatory cytokines were reduced dramatically by CUR and could suppress the PMMA particle-induced inflammation (Figure 6). Thus, our results indicate that CUR can be a therapeutic agent for wear particle-induced AL.

## Figures and Tables

**Table 1:** Effect of curcumin on liver and kidney functions of mice after surgery (*n* = 12).

	Liver function		Kidney function	
	ALT (U/l)	AST (U/l)	Cre (*μ*mol/l)	BUN (nmol/l)
PBS group	21.98 ± 3.43	17.56 ± 2.83	35.62 ± 2.84	5.72 ± 1.22
CUR group	21.06 ± 3.75	16.97 ± 2.53	34.52 ± 2.53	5.35 ± 1.02
